# In this month’s Bulletin

**DOI:** 10.2471/BLT.23.000123

**Published:** 2023-01-01

In the editorial section, Etienne V. Langlois et al. (2) argue for better postnatal care services. Karen I. Barnes et al. (3) describe the contributions made to clinical pharmacology by Peter Ian Folb.

Tatum Anderson (6–7) reports on emergency response initiatives in Botswana, Mauritania, Niger, Nigeria and Togo. Anthony Fauci talks to Gary Humphreys (8–9) about his work with the National Institute of Allergy and Infectious Diseases. 

## Bangladesh, Benin, Burkina Faso, Burundi, Chad, Democratic Republic of the Congo, Ethiopia, Ghana, Indonesia, Islamic Republic of Iran, Jordan, Kenya, Liberia, Malawi, Mali, Morocco, Myanmar, Senegal, South Africa, Sri Lanka, Sudan, Uganda, United Republic of Tanzania, Zimbabwe

### Mother and child deaths

Merlin L. Willcox et al. (62–75) review characteristics of mortality surveillance.

## India

### Optimal use of antimicrobials 

Sonam Vijay et al. (20–27) track stewardship in tertiary hospitals.

### Tuberculosis treatment success

Rajaram Subramanian Potty et al. (28–35) assess support group participation.

## Rwanda

### Accurate diagnosis of breast cancer

Parsa Erfani et al. (10–19) compare costs of biomarker analysis methods.

## Global

### Rational use of medications

Loai Albarqouni et al. (36–61) quantify the extent of unnecessary treatments.

